# Everyday Beliefs About Emotion Perceptually Derived From Neutral Facial Appearance

**DOI:** 10.3389/fpsyg.2020.00264

**Published:** 2020-02-28

**Authors:** Daniel N. Albohn, Reginald B. Adams

**Affiliations:** Department of Psychology, The Pennsylvania State University, University Park, PA, United States

**Keywords:** facial expression, person perception, neutral, neutral face, impression formation

## Abstract

The evolution of the human brain and visual system is widely believed to have been shaped by the need to process and make sense out of expressive information, particularly via the face. We are so attuned to expressive information in the face that it informs even stable trait inferences (e.g., Knutson, 1996) through a process we refer to here as the *face-specific fundamental attribution error* (Albohn et al., 2019). We even derive highly consistent beliefs about the emotional lives of others based on emotion-resembling facial appearance (e.g., low versus high brows, big versus small eyes, etc.) in faces we know are completely devoid of overt expression (i.e., emotion overgeneralization effect: see Zebrowitz et al., 2010). The present studies extend these insights to better understand lay beliefs about older and younger adults’ emotion dispositions and their impact on behavioral outcomes. In Study 1, we found that older versus younger faces objectively have more negative emotion-resembling cues in the face (using computer vision), and that raters likewise attribute more negative emotional dispositions to older versus younger adults based just on neutral facial appearance (see too Adams et al., 2016). In Study 2, we found that people appear to encode these negative emotional appearance cues in memory more so for older than younger adult faces. Finally, in Study 3 we exam downstream behavioral consequences of these negative attributions, showing that observers’ avoidance of older versus younger faces is mediated by emotion-resembling facial appearance.

## Introduction

That humans possess theory of mind–the ability to read others to make accurate assessments of others’ seemingly invisible internal states–is widely hailed as evidence that the evolution of the human brain, and visual system in particular, has been shaped by a need to process and derive social meaning from others’ expression, particularly via the face (Allison et al., 2000; Emery, 2000).

As humans we are so tuned to reading expressive information from others that we fall prey to what we will refer to here as *face-specific fundamental attribution errors* (Albohn et al., 2019). Just like the classic *fundamental attribution error*, which posits that individuals tend to ascribe internal and stable traits based solely on external features, individuals tend to ascribe enduring personality traits and emotional dispositions to others based on their overt facial expressions (see also, Knutson, 1996; Hess et al., 2000). We are so tuned to reading expressive information from the face that even when there is no expressive information present individuals base their beliefs about others’ emotional dispositions on emotion-resembling appearance cues in the face (i.e., emotion overgeneralization; Zebrowitz et al., 2010). Here we argue that the mechanism underlying emotion overgeneralization is a face-specific fundamental attribution error. That is, individuals overgeneralize emotions *because* they are using facial appearance cues that resemblance expressions to make their judgments about enduring impressions of others.

Such appearance cues have been argued to contribute to various emotion stereotypes. For example, male faces (versus female faces) tend to structurally resemble anger expressions with lowered brows, thin lips, and square jaws, whereas female faces structurally resemble happy faces, in line with prevailing gender emotion stereotypes (see Adams et al., 2015 for review; Hess et al., 2004; Becker et al., 2007; Zebrowitz et al., 2010; Palumbo et al., 2017). Further, as a face ages, it takes on more emotion-resembling cues (Malatesta et al., 1987; Adams et al., 2016), which have been argued to contribute to negative age-related stereotypes (Hess et al., 2012).

A large meta-analytic review (Kite et al., 2005) and a study of 26 different cultures revealed strong evidence for negative age-related stereotypes (Löckenhoff et al., 2009). Critically, there is evidence that this bias is largely linked to impressions derived from faces. For instance, when asked to rate impressions of a “typical” younger and older person when not viewing faces, the typical negative bias disappeared (Boduroglu et al., 2006). This latter finding suggests a perceptual basis for age-related negative stereotypes, one we argue here is related to age-related emotion-resembling cues in the face. The most prevalent age-related stereotype is that older people are more prone to negative emotion than their younger counterparts, which arguably directly contributes to a general negativity bias (Fabes and Martin, 1991; Kite et al., 2005; Ebner, 2008).

## The Current Work

We first report a preliminary study to demonstrate lay beliefs about how informative neutral faces are to individuals. We hypothesized that participants would report that neutral faces offer little-to-no information when deriving emotional beliefs about others. We do this first to contrast with our subsequent studies in which we aimed to show that neutral faces are indeed utilized to form emotional impressions of others.

Next, in Study 1 we subjected all neutral faces to a computer vision analysis designed to read emotion from faces to establish objective evidence (from pixel and facial metric data) that there are more negative emotion cues in older versus younger faces. Then we had observers rate older and younger neutral faces on an emotion disposition profile to examine the influence of aging on everyday beliefs about emotions people report when rating faces. We predicted that there would be a bias to rate older faces as more likely to experience negative emotions, as prior work has suggested that aging cues in the face resemble negative emotions such as sadness and anger (e.g., Ebner, 2008; Hess et al., 2012), which we argue here in turn contributes to negative age-related stereotypes and bias.

Next, in Study 2 we examined people’s mental representations of older versus younger neutral faces to see if the images people hold in their memory of “typical” faces contain emotional tone. Assuming that people’s mental representations of older and younger faces reflects that which has been previously seen and stored in memory, we hypothesized that composites generated of older versus younger neutral faces, using a reverse correlation (RC) task (Dotsch et al., 2008), would be rated as higher on negative emotions by independent raters.

Finally, in Study 3 we examined the potential consequences of these negative age-related everyday beliefs about emotion on motivated behavior. Utilizing a modified approach/avoid task, we predicted that older faces would be avoided more than younger faces regardless of overt expression, and that older neutral faces would be avoided at a similar level as negative expressions due to emotion resemblance of age-related cues in the face. This latter prediction was tested using mediational analyses to show that age influences avoidance via emotion-resembling cues in the face.

## Preliminary Study: Everyday Beliefs About Neutral Displays

### Methods

#### Participants

Participants (*N* = 32; 22 females, 10 males, *M*_*age*_ = 18.78) were college students and received course credit in exchange for participation.

#### Stimuli and Procedure

Participants were asked “How socially informative is a/an (younger/older) (male/female) neutral face?” for a total of four trials per participant.

Each statement was presented in random order across participants. Participants were allowed to type their response in a text box. At the beginning of the experiment they were asked to provide at least one sentence per question. After each free response, participants were asked the same question but asked to provide a numerical value on a Likert-type scale with anchors 1 = “Nothing at all” to 7 = “A lot.”

### Results

Overall, across both older and younger adults the modal response on the “social informativeness” of a neutral face was two, suggesting that the majority of participants believed that neutral faces contained very little useful information for making judgments.^1^

Next, we conducted a two (gender: male, female) by two (age: old, young) linear mixed effects model to examine whether participants’ ratings of neutral faces varied by age or gender. Our linear mixed effects model contained random intercepts for participants.

There was only a main effect of age, *F*(1, 96) = 6.60, *p* = 0.012. Overall, participants believed that older adult neutral faces [estimated marginal mean (EMM) = 3.14] provided significantly less information than younger adult neutral faces (EMM = 3.73).

We also analyzed our open-ended data with a thematic analysis, which revealed results similar to our rating data. The full linear mixed model, along with additional thematic analysis, is provided in Supplementary Materials 1.

In sum, this pilot study suggests lay beliefs regarding neutral faces are that they are relatively uninformative, particularly older adult neutral faces. We present this data first because although everyday beliefs about neutral faces may be that they convey little information, in the subsequent studies we aim to demonstrate that they nonetheless contribute greatly to everyday beliefs about emotion, and of particular relevance to the current work, age-related beliefs.

## Study 1: Demonstration of Age-Related Emotion Overgeneralization

Study 1 was designed to demonstrate age-related emotion overgeneralization when making judgments of intentionally posed neutral faces. Specifically, we predicted that individuals would differentially attribute enduring emotional dispositions more so to older and younger adult neutral faces despite the lay belief that neutral faces provide little useful information.

### Methods

#### Participants and Stimuli

Participants (*N* = 49; 39 females, 10 males, *M*_*age*_ = 19.2) were college students. Stimuli were 888 older and younger adult neutral faces from the FACES image set (Ebner et al., 2010), the Face Database (Minear and Park, 2004), and the Humboldt image set (see, Adams et al., 2016) for a total of 394 old female adults, 209 old male adults, 144 young female adults, and 141 young male adults.

#### Procedure

Participants were instructed that they would be shown faces of individuals and asked to rate them on “how likely each individual is to feel the following emotions,” and then each emotion was listed in the following order: anger, joy, disgust, sadness, fear, and surprise. Next, individuals randomly saw 100 images from the image pool along with rating sliders for each of the six emotions. Each rating slider was anchored with points 1 = “Not at all,” 4 = “Somewhat,” and 7 = “Very much.”

### Results

#### FaceReader Results

To first examine whether the observer rating results were driven by misconceptions of emotion cues in the aging neutral face, we analyzed each face utilizing FaceReader^TM^ 6.1 (Noldus, 2015). FaceReader^TM^ is a commercial computer vision tool used to objectively analyze the presence of expressions, action units, and emotional overall valence in facial images. FaceReader^TM^ is well established and validated in the scientific literature, with results approaching expert level (Lewinski, 2015; Adams et al., 2016).

Each face was analyzed using FaceReader^TM^ 6.1’s general model. As part of the general model, FaceReader^TM^ outputs a valence score between −1 and +1 that corresponds to the amount of predicted negativity or positivity, respectively, present in each face. We analyzed each of the neutral faces’ valence score to compare whether older adult neutral faces were objectively categorized as more negative than younger adult neutral faces. Of the 888 faces, 878 face images were recognized and able to be computed with FaceReader’s^TM^ detection algorithm. In line with our hypothesis, the valence of older adult neutral faces (EMM = −0.051) was more negative than younger adult neutral faces (EMM = 0.000), *F*(1, 877) = 14.14, *p* < 0.001, *R*^2^ = 0.02.

#### Rating Results

##### Preprocessing

To prepare the data for analysis, we first removed outliers. Outliers that were 1.5 times below or above the interquartile range were removed. Two hundred and twenty-one (0.004%) responses were removed in this manner. We conducted a 2 (image age: old, young) by 6 (emotion rating: angry, disgust, joy, fear, neutral, and surprise) linear mixed effects model with fixed effects for image age and emotion rating. We included random intercepts for each participant and image, and random slopes for image age within participant.

##### Analysis

There was a main effect for emotion rating, *F*(5, 28230.2) = 130.96, *p* < 0.001. There was also a main effect of image age, *F*(1, 59.80) = 9.39, *p* = 0.003. Overall, older adult images (EMM = 2.80) were rated higher on emotionality compared to younger adult images (EMM = 2.71), *t*(29127) = 3.07, *p* = 0.002.

Critically, these main effects were qualified by an interaction between emotion rating and image age, *F*(5, 28230.3) = 12.71, *p* < 0.001. On average, older adults were expected to feel more anger, disgust, and surprise compared with younger adults. Means and pairwise comparisons are reported in Table 1.

**TABLE 1:** Pairwise comparisons for emotion ratings between young and old faces for Study 1.

Rating	Older adult EMM	Younger adult EMM	Estimate	*t*-value	*p*-value
Anger	2.94	2.75	0.19	3.65	<0.001
Disgust	2.99	2.63	0.36	6.88	<0.001
Fear	2.50	2.50	–0.002	–0.05	0.963
Joy	3.02	3.00	0.02	0.31	0.755
Sad	2.94	3.00	–0.06	–1.17	0.244
Surprise	2.48	2.36	0.12	2.38	0.018

Additionally, we tested whether each images’ valence score mediated the relationship between image age and average participant rating score. FaceReader^TM^ valence mediated this relationship for all emotion ratings except surprise and fear (all significant indirect effects *p*’s < 0.001). The full linear mixed effects model, and mediation analyses are presented in Supplementary Materials 2.

In sum, Study 1 shows that participants derive beliefs about the emotional dispositions of younger and older adults based solely on their neutral faces. Overall more emotion is perceived in older faces, particularly more anger and disgust, presumably due to age-related appearance. That we found no difference in sadness and greater surprise for older adults, is perhaps due to the fact that we were using a very large, naturalistic data set of neutral faces. However, more overall affective negativity was detected by FaceReader^TM^ based solely on objective facial cues, which in turn mediated participant responses, underscoring emotion resemblance as a primarily contributing influence in these everyday beliefs regarding age-related emotional dispositions.

## Study 2: Bias in Mental Representations of Aged Faces

Study 2 was designed to examine how older and younger faces are encoded in memory. To do this we used a RC procedure (Dotsch et al., 2008) to generate composite images that reflect “mind’s eye” representations of a typical older versus younger adult. Study 1 demonstrated that older versus younger neutral faces objectively contain more negative emotion cues and are subjectively rated as expected to experience more negative emotions (particularly anger and disgust). Study 2 examined whether these age-related negative emotion-resembling appearance cues are also encoded into memory.

### Method

#### Participants

Twenty-seven participants (16 females, 11 males) created RC classification images (CIs), and 66 participants^2^ (46 females, 20 males) rated each image. Participants were college students that participated in exchange for course credit.

#### Study Stimuli

Stimuli for this study were created following the typical RC procedure. Briefly, 300 image pairs were created by overlaying random sinusoidal noise or the inverse of the random noise pattern atop an age-ambiguous base image created by averaging old and young, male and female neutral faces from the Ebner face set (Ebner et al., 2010) together (see, Dotsch et al., 2008 for full RC method).

**FIGURE 1:**
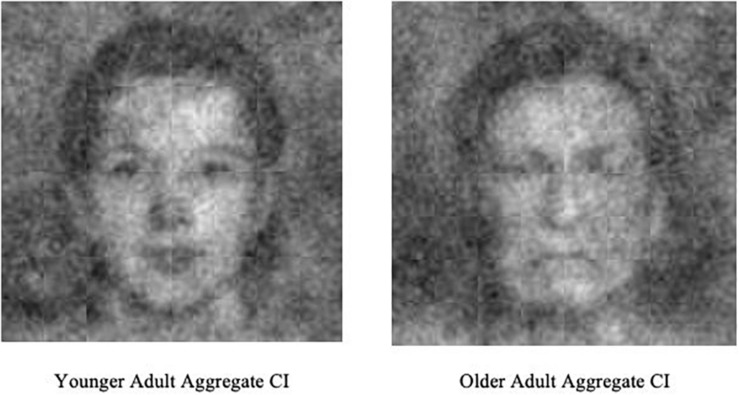
Example classification images (CIs) produced across all participants for Study 2.

On each of the 300 trials, participants were asked to select between the image pairs the one that “looked most like a typical (older/younger) adult.” Participants completed this procedure for both age blocks (old/young), which were randomized between participants. Then, by aggregating participant responses across each trial and block, a meaningful representation of what the individual was imagining when thinking of the age group emerges from the random noise (see Figure 1 for examples). We collected 56 stimuli in this manner (27 participants × 2 blocks + 2 aggregate images).

#### Rating Procedure

Participants were instructed that they would be shown faces that “had been manipulated using a computer program,” and to rate each face on several emotions/traits. Next, participants saw each of the 56 stimuli individually and asked to rate it on how angry, disgustful, fearful, feminine, happy, masculine, neutral, sad, and surprised it appeared before moving on to the next image. Images were randomized between participants. Each Likert-type scale was anchored with points 1 = “Not at all,” 4 = “Somewhat,” and 7 = “A lot.”

### Results

We conducted a 2 (image age: older, younger) by 8 (rating: angry, disgustful, fearful, feminine, happy, masculine, neutral, sad, and surprised) linear mixed effects model with fixed effects for image age and emotion rating. We included random intercepts for each participant and image. Ratings of aggregate CIs are reported in Supplementary Materials 3.

There was a main effect for emotion rating, *F*(8, 31338.2) = 731.043, *p* < 0.001. Of note, CIs were rated highest for appearing neutral, and neutral ratings were significantly higher than all other emotion ratings. There was also a main effect for image age, *F*(1, 52.1) = 18.99, *p* < 0.001. On average, older adult CI images (EMM = 3.17) were rated higher than the younger adult CI images (EMM = 3.03), *t*(31473) = 4.36, *p* < 0.001.

There was also an interaction between emotion rating and image age, *F*(8, 31338.2) = 92.07, *p* < 0.001. Overall, older adult CIs were rated as expressing more anger, disgust, fear, sadness, and less happiness and neutrality than younger adult CIs (Table 2 reports full pairwise comparisons). The full linear mixed effects model is presented in Supplementary Materials 4.

**TABLE 2:** Pairwise comparisons for Study 2.

Rating	Older adult EMM	Younger adult EMM	Estimate	*t*-value	*p*-value
Anger	3.32	2.38	0.95	15.57	<0.001
Disgust	3.16	2.54	0.62	10.32	<0.001
Fear	2.75	2.55	0.19	3.22	0.001
Joy	1.92	2.75	–0.83	–13.80	<0.001
Sad	3.58	3.04	0.54	9.05	<0.001
Surprise	2.02	2.11	–0.09	–1.51	0.132
Neutral	4.07	4.17	–0.10	–1.66	0.097
Feminine	3.71	3.70	0.01	0.16	0.873
Masculine	3.97	3.98	–0.01	–0.17	0.864

In sum, Study 2 demonstrated that individuals hold internal representations of typical aged faces that contain more negative emotionality than younger faces. Because internal representations for groups are largely the product of what has been experienced or seen before, these results suggest that the negativity “read into” aged faces are perceptually encoded.

## Study 3: Consequences of Perceiving Emotional Negativity in Older Neutral Faces

Study 3 examines a potential consequence of perceiving older neutral faces as expressing negative affect. Specifically, we predicted that older adult neutral faces would be avoided to a greater extent than younger adult neutral faces, and that older adult neutral faces would be avoided in a manner similar to other negative emotions.

### Method

#### Participants and Stimuli

Participants (*N* = 52; females = 19, males = 32, *M*_*age*_ = 19.26) were college students.

Stimuli were 575 old and young adult emotional (angry, fear, joy, sad) and neutral faces from the FACES image set (Ebner et al., 2010) for a total of 174 old female adults, 150 old male adults, 174 young female adults, and 174 young male adults. We included expressive faces to compare neutral face responses to positive and negative expressive faces.

#### Procedure

Participants were instructed to imagine for each trial (face) that they were in a digital face-to-face meeting (e.g., Skype) with the individual presented. Participants were then told that for each trial they should use the mouse to place the individual presented at a distance that they felt comfortable interacting with that person. The experimental stimulus size was mapped to the participant’s mouse movements such that pushing the mouse upward (away) made the image smaller, and thus appear as if it were further in the distance. Likewise, pulling the mouse downward (toward) made the image larger, and thus appear as if it were closer. Each trial started with the image presented focally but at a random distance (size). This procedure is a modified approach-avoid task whereby participants get stimulus-level feedback during each trial (see, Phaf et al., 2014).

During each trial one of five random hallway backdrops appeared behind each image to add to the illusion of depth. Background images had no effect on the results, and thus were collapsed during analysis, *F*(4, 3860.5) = 1.18, *p* = 0.32. In-between each mouse movement trial there was a 200 ms fixation dot. Participants completed 100 trials and randomly saw 100 images from the total pool of images. On average, participants saw approximately 5.2 (*SD* = 0.26) images from each emotion by image gender by image age category.

### Results

In order to fully explore the relationship between emotion, age, and approach/avoidant behavior, we first analyzed participant-level data for each age group and emotion expression. Following this, we analyzed the results at the stimulus-level using a mediation to examine the effect of each stimulus’ likelihood of expressing a given emotion on approach/avoidant behavior in relation to age of the stimulus itself.

#### Avoidant Behavior

We conducted a two (image age: old, young) by five (image emotion: angry, fear, happy, neutral, sad) linear mixed effects model with fixed effects for image age and image. We included random intercepts for each participant and image, and random slopes within image age group. The scale factor for the image (smaller values = stimuli placed farther away) was used as the dependent variable for all analyses.

**FIGURE 2:**
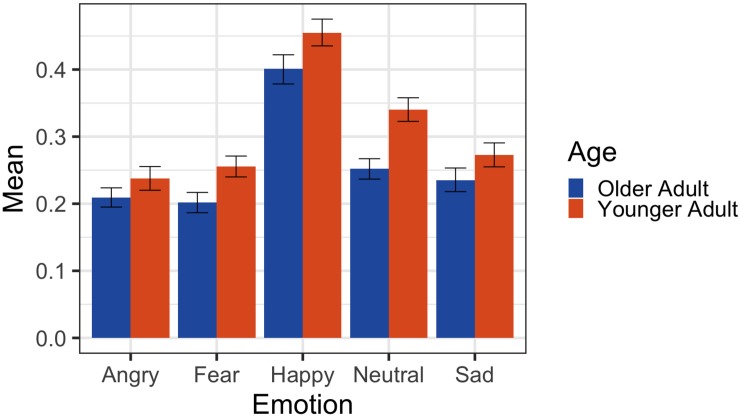
Participant response means and 95% CIs for Study 3 approach/avoid mouse placement task. *Y*-axis represents relative size of the stimulus, with smaller values indicating the stimulus being placed further away. Placement values range from 1 (largest size) to 0 (smallest size).

There was a main effect for image emotion, *F*(4, 519.37) = 213.35, *p* < 0.001. There was also a main effect of image age, *F*(1, 47.33) = 41.75, *p* < 0.001, such that older adult images (EMM = 0.26) were placed further away compared to young adult images (EMM = 0.31), *t*(4200) = −6.46, *p* < 0.001. There was also an image emotion by image age interaction, *F*(4, 519.16) = 3.39, *p* < 0.001 (see Figure 2).

There was also an image emotion by image age interaction, *F*(4, 519.16) = 3.39, *p* < 0.001. *Post hoc* analysis of this interaction revealed that across all emotion types older adults were placed farther away than younger adults, *p*’s = 0.02–0.0001. Of the five emotions that participants saw, neutral faces showed the largest difference between old (EMM = 0.25) and young (EMM = 0.33) faces, *t*(4200) = −6.55, *p* < 0.001. Indeed, participants’ responses to older adult neutral stimuli were significantly more similar to negative emotion faces [*r*(40) = 0.86, *p* < 0.001] than they were to positive emotion faces [*r*(40) = 0.38, *p* < 0.001], *z* = 4.65, *p* < 0.001. Participant responses to young adult neutral faces followed a similar pattern, with a higher correlation with negative expressions [*r*(40) = 0.65, *p* < 0.001] than with positive expressions [*r*(40) = 0.38, *p* = 0.012]. Critically, however, the difference between these two similarity correlations only approached significance, *z* = 1.84, *p* = 0.07. Additionally, when we controlled for differences in approach/avoidant behavior between old and young expressive faces, there was still a significant difference between the distance participants placed older adult neutral faces compared to younger adult neutral faces, *F*(1, 66.44) = 30.28, *p* < 0.001.

Together, these results suggest that while there may be a general negativity bias toward older adults, this bias alone cannot fully explain the large differences observed for placement of neutral faces. The full linear mixed effects model and all of the pairwise comparisons are reported in Supplementary Materials 5.

#### Stimulus-Level Characteristics

While our participant-level data suggests that there are nuanced differences for older adult neutral faces, we wanted to further examine the causal effects of stimulus-level characteristics on approach/avoidance behavior. We computed a single negativity index by taking the averaged emotion scores (positive emotions reverse scores) provided by participants in Study 1 for each image used in Study 3 and summing. We then conducted a simple mediation to evaluate whether the negativity index mediated the relationship between stimulus age (old versus young) and distance participants placed the neutral face image (approach/avoid behavior). In line with our hypothesis, greater perceived negative emotional disposition on older adult neutral faces mediated the relationship between stimulus age and placing those faces further away. The standardized indirect effect was 0.04, and was significant with 10,000 bootstrapped samples, *F*(2, 112) = 31.34, *p* < 0.001, *R*^2^ = 0.36, CIs [−0.1, 0.1] (see also Supplementary Figure S1).

## General Discussion

Across three studies we presented data that shows the inherent compulsion of observers to perceive more negative emotion in non-expressive age-related appearance. In a preliminary study we showed that participants believed that neutral faces of all age groups provided little useful information, but in particular older adult neutral faces. Despite this, in Study 1 when participants were presented with older and younger adult neutral faces and asked to rate enduring emotion dispositions, observers consistently reported that older adults were expected to experience more negative emotions (e.g., anger, disgust) and surprise. This is likely due to older adult neutral faces containing more aging cues that can be misinterpreted as emotion cues, which was underscored by an objective computer vision approach also reading relatively more negative affect in older versus younger neutral faces. This result conceptually replicates previous research that has found a similar effect for perceptually based negative, age-related stereotypes, while also extending it to enduring emotion dispositions regarding age-related, everyday beliefs about emotional experience (Hess et al., 2012; Adams et al., 2016).

In Study 2 we found that participants also hold internal mental representations of “typical” older neutral faces that–although rated as appearing neutral–contain more negativity than younger adult internal representations. Given evidence in Study 1 that older faces objectively contain more negative emotion-resembling cues than younger faces, Study 2 goes one step further to show that these cues appear to be encoded into memory becoming part of one’s facial aging prototype.

Lastly, in Study 3 we examined one potential consequence that these everyday beliefs about age and perceived emotion cues in a neutral face can have on real-world behavior. Utilizing a modified approach/avoid task whereby the participant must place an older or younger adult face either closer or further away, participants consistently and across all emotions (including neutral) placed older adults further away, suggesting a tendency to avoid. Importantly, the largest of the observed effects was for neutral faces, and overall emotional disposition negativity mediated the relationship between stimulus age and the distance at which neutral faces were placed.

It is important to note that despite our mediational evidence showing that observers use negative emotion appearance to make judgments of older adults’ neutral faces, there are likely other contributing mechanisms at play as well. In particular, the results may also be in part due to an in-group bias, or due to some other characteristic about older adult neutral faces, such as resemblance to anomalous faces or attractiveness. Indeed, work has shown that both resemblance to anomalous faces and attractiveness mediated the relationship between age and negative traits (Zebrowitz et al., 2003; Palumbo et al., 2017). In our studies, all of our participants were college-aged students rating faces of younger and older adults. While it is certainly possible that an in-group bias may be contributing to our observed effects, our data suggest that negative emotion appearing cues are a large contributor. Indeed, two mediation models show that objective negative valence (Study 2) and perceived negative emotion disposition (Study 3) mediate (negative) participant responses to older adult faces.

In sum, we provide evidence that individuals ascribe enduring emotion traits as a function of age based on the physical appearance of an actor with a neutral visage. Importantly, engaging in a face-specific fundamental attribution error influences everyday beliefs about the emotional lives of older versus younger adults, and in turn has real behavioral consequences, such as a tendency to avoid actors that present with neutral displays that contain more negative-appearing face cues.

That individuals are so tuned to extract any socially relevant and useable information from even a non-expressive face emphasizes just how important it is to understand what cues observers utilize from the face to form beliefs about the individual, and the consequences that these beliefs have on real-world behavior (regardless of their accuracy). Indeed, the way in which we form everyday beliefs about an individual (via emotion cues) can have a profound impact on behavior, and in the case of aging, negative, avoidance-related consequences.

## Data Availability Statement

The datasets generated for this study are available on request to the corresponding author.

## Ethics Statement

The studies involving human participants were reviewed and approved by the Office for Research Protections Pennsylvania State University. Written informed consent for participation was not required for this study in accordance with the National Legislation and the Institutional Requirements.

## Author Contributions

DA and RA contributed to the conception and design of the studies and contributed to writing, revising, and approving the manuscript. DA ran the studies and analyzed the data under the supervision of RA.

## Conflict of Interest

The authors declare that the research was conducted in the absence of any commercial or financial relationships that could be construed as a potential conflict of interest.

## Footnotes

1 Modal response was two rather than one most likely due to scale extremity response bias (i.e., avoiding the very lowest or highest points of the scale).

2 Six participants did not complete the experiment. However, including them did not change the results and are thus included in all analyses presented.
