# Supplementary material for: Everyday Beliefs About Emotion Perceptually Derived From Neutral Facial Appearance
Source: Front Psychol. 2020 Feb 28;11:264. doi: 10.3389/fpsyg.2020.00264 (PMC7059455; doi:10.3389/fpsyg.2020.00264)
Supplement: Supplementary file 1 [file Data_Sheet_1.docx]

**Supplemental Materials for:**

Everyday Beliefs About Emotion Perceptually Derived from Neutral Facial Appearance

Daniel N. Albohn & Reginald B. Adams, Jr.

The Pennsylvania State University

| **Random Effects** | | |
| --- | --- | --- |
| **Groups Name** | **Variance** | **Std. Dev** |
| Participant (intercept) | 0.72 | 0.85 |
| Residual | 1.71 | 1.31 |
| Number of obs: 128, groups: participants, 33 | | |

**Supplemental Materials 1**

| **Fixed Effects** | | | | | |
| --- | --- | --- | --- | --- | --- |
|  | **Estimate** | **Std. Error** | **t value** | **df** | **p-value** |
| Intercept | 3.28 | 0.28 | 22.74 | 101.41 | < .001 |
| Gender (male) | -0.28 | 0.33 | -0.86 | 96 | .392 |
| Age (young) | 0.53 | 0.33 | 1.63 | 96 | .107 |
| Gender*age | 0.13 | 0.46 | 0.27 | 96 | .787 |

***Table S1a. Full Linear Mixed-Effects Model for Pilot Study rating results.***

**Thematic Analysis**

Having established that individuals report that older adult neutral faces provide less information than younger adult neutral faces, we sought to examine what themes emerged across participants’ open-ended responses to the information that neutral faces convey. The authors categorized each of the neutral open-ended responses into broad categories. A total of six categories emerged (along with an unknown category; See Table S1b for categories and examples).

| **Category** | **Example Response** | **Proportion Present** | | |
| --- | --- | --- | --- | --- |
|  |  | **Older Adult (*N* = 63)** | **Younger Adult (N = 64)** | |
| Nothing, very little, neutral | “Not informative at all. None[.]” | 0.44 | | 0.38 |
| Negative emotion | “A neutral face can sometimes give off an angry impression or like the girl is mad. This is pretty common among young female emotionless faces.” | 0.11 | | 0.06 |
| Positive emotion | “[V]ery informative, positive expressions[.]” | 0.08 | | 0.09 |
| Mood, feelings, general emotion | “Can tell a little bit. Can tell his mood[.]” | 0.06 | | 0.09 |
| Physical features (wrinkles) or demographics (age, race) | “I can only infer the gender, but the age is a little less subjective if we are referring to young adults.” | 0.05 | | 0.11 |
| Internal states other than emotion, thinking | “That the man is either tired or bored.” | 0.16 | | 0.23 |
| Unknown/Other | “Aging experience[.]” | 0.10 | | 0.03 |

***Table S1b. Themes and examples from participants’ free responses to the question “What information can be extracted from a neutral face?” for Study 1 thematic analysis.***

To test if there was a statistical difference in the proportions of the themes we observed in the open-ended responses, we counted the number of each theme present across each age, gender, and category. We then subjected our count data to a Poisson regression with theme, age, gender, and their interaction as parameters in the model. The only statistically significant effect was a main effect of theme type, $\chi^{2}$(6) = 66.59, *p* < .001. Post hoc pairwise analyses revealed that the theme of “nothing, very little, neutral” appeared more than all of the other six types of themes, though this did not vary by age, $\chi^{2}$(6) = 6.37, *p* = .383, or by gender, $\chi^{2}$(6) = 4.92, *p* = .554. The pairwise analyses for the significant main effect is displayed in Table S1c.

| **Rating Contrast** | **Estimate** | ***t* value** | ***p* value** |
| --- | --- | --- | --- |
| **“Neutral, very little…” – “Negative emotion”** | 1.59 | 4.63 | < .001 |
| **“Neutral, very little…” – “Positive emotion”** | 1.55 | 4.67 | < .001 |
| **“Neutral, very little…” – “Mood, feelings, …”** | 1.67 | 4.74 | < .001 |
| **“Neutral, very little…” – “Physical features, …”** | 1.73 | 4.66 | < .001 |
| **“Neutral, very little…” – “Internal states, …** | 0.75 | 3.03 | .039 |
| **“Neutral, very little…” – “Unknow/other”** | 2.01 | 4.67 | < .001 |

***Table S1c. Pairwise comparisons for the Pilot Study between themes of “Neutral, very little, or nothing” and all other themes.***

**Supplemental Materials 2**

| **Random Effects** | | | |
| --- | --- | --- | --- |
| **Groups Name** | **Variance** | **Std. Dev** | **Cor** |
| Participant (intercept) | 1.02 | 1.01 |  |
| Image | 0.02 | 0.16 |  |
| Age | 0.03 | 0.18 | 0.19 |
| Residual | 1.95 | 1.40 |  |
| Number of obs: 29127, groups: image, 888; participants, 49 | | | |

| **Fixed Effects** | | | |  |  |
| --- | --- | --- | --- | --- | --- |
|  | **Estimate** | **Std. Error** | **t value** | **df** | **p-value** |
| Intercept | 2.94 | 0.15 | 20.07 | 50.48 | < .001 |
| Rating (disgust) | 0.05 | 0.03 | 1.47 | 28230 | .141 |
| Rating (fear) | -0.44 | 0.03 | -12.91 | 28230 | < .001 |
| Rating (joy) | 0.08 | 0.03 | 2.30 | 28230 | .022 |
| Rating (sad) | 0.00 | 0.03 | 0.12 | 28230 | .908 |
| Rating (surprise) | -0.46 | 0.03 | -13.29 | 28230 | < .001 |
| Age (young) | -0.19 | 0.05 | -3.65 | 327.7 | < .001 |
| Rating (disgust) * age (young) | -0.17 | 0.06 | -2.75 | 28230 | .006 |
| Rating (fear) * age (young) | 0.19 | 0.06 | 3.15 | 28230 | .002 |
| Rating (joy) * age (young) | 0.17 | 0.06 | 2.84 | 28230 | .005 |
| Rating (sad) * age (young) | 0.25 | 0.06 | 4.10 | 28230 | < .001 |
| Rating (surprise) * age (young) | 0.07 | 0.06 | 1.09 | 28230 | .275 |

**Table S2a. Full Linear Mixed-Effects Model for Study 1.**

**Mediation Analyses for Study 2**

We conducted a series of simple mediations to assess whether the objective valence score computed by FaceReader mediated the relationship between image age and average participant response. Valence mediated the relationship between image age and participant response for anger, disgust, sad, and joy. Table S2c presents the results of the six mediation analyses.

|  | **Total Effect** | | | **Indirect Effect** | |
| --- | --- | --- | --- | --- | --- |
| **Emotion** | **Estimate** | **R^2^** | **95% CI** | **Estimate** | **95% CI** |
| **Anger** | -0.13^+^ | 0.004 | [-0.27, 0.01] | -0.05*** | [-0.07, -0.02] |
| **Disgust** | -0.33*** | 0.03 | [-0.46, -0.19] | -0.03*** | [-0.05, -0.01] |
| **Fear** | -0.07 | 0.001 | [-0.18, 0.05] | -0.01 | [-0.02, 0.01] |
| **Joy** | -0.06 | 0.001 | [-0.23, 0.11] | 0.08*** | [0.04, 0.12] |
| **Sad** | 0.06 | 0.001 | [-0.07, 0.19] | -0.02*** | [-0.04, -0.01] |
| **Surprise** | -0.19*** | 0.01 | [-0.31, -0.07] | 0.01 | [-0.01, 0.03] |

***Table S2b. Mediation analyses of valence on the relationship between image age and emotion ratings. Older adults acted as the control group and younger adults the treatment.***

**Supplemental Materials 3**

We analyzed the ratings to aggregate CI images separately from individual CIs. Similar to ratings of the individual CIs, we conducted a 2 (image age: older, younger) by 8 (rating: angry, disgustful, fearful, feminine, happy, masculine, neutral, sad, and surprised) linear mixed effects model with fixed effects for image age and emotion rating. We included random intercepts for each participant.

There was a main effect for emotion rating, *F*(8, 1096.6) = 33.62, *p* < .001. There was also a main effect for image age, *F*(1, 1104.0) = 22.94, *p* < .001. On average, the aggregate older adult CI (EMM = 3.32) were rated higher than the aggregate younger adult CI (EMM = 2.91), *t*(41589) = 1.93, *p* = .053.

There was an interaction between emotion rating and image age, *F*(8, 1096.6.2) = 50.06, *p* < .001. Overall, older adult CIs were rated as expressing more anger, disgust, sadness, and less happiness and neutrality than younger adult CIs (Table S3a reports full pairwise comparisons). The full linear mixed effects model is presented in Table S3b.

| **Rating** | **Older Adult EMM** | **Younger Adult EMM** | **Estimate** | ***t* value** | ***p* value** |
| --- | --- | --- | --- | --- | --- |
| **Anger** | 5.64 | 1.95 | 3.68 | 11.35 | < 0.001 |
| **Disgust** | 4.41 | 2.15 | 2.26 | 6.97 | < 0.001 |
| **Fear** | 2.42 | 2.13 | 0.29 | 0.89 | 0.371 |
| **Joy** | 1.12 | 3.89 | -2.77 | -8.54 | < 0.001 |
| **Sad** | 3.47 | 2.36 | 1.11 | 3.42 | < 0.001 |
| **Surprise** | 1.74 | 2.04 | -0.31 | -0.95 | 0.343 |
| **Neutral** | 3.23 | 3.96 | -0.73 | -2.25 | 0.025 |
| **Feminine** | 3.90 | 3.98 | -0.08 | -0.24 | 0.808 |
| **Masculine** | 3.94 | 3.68 | 0.27 | 0.82 | 0.410 |

***Table S3a. Pairwise comparisons for Study 2.***

| **Random Effects** | | |
| --- | --- | --- |
| **Groups Name** | **Variance** | **Std. Dev** |
| Participant (intercept) | 0.09 | 0.31 |
| Residual | 2.19 | 1.48 |
| Number of obs: 1179, groups: participants, 66 | | |

| **Fixed Effects** | | | |  |  |
| --- | --- | --- | --- | --- | --- |
|  | **Estimate** | **Std. Error** | **t value** | **df** | **p-value** |
| Intercept | 5.64 | 0.19 | 1128.65 | 30.03 | < .001 |
| Rating (disgust) | -1.22 | 0.26 | 1096.59 | -4.71 | < .001 |
| Rating (fear) | -3.22 | 0.26 | 1096.59 | -12.38 | < .001 |
| Rating (feminine) | -1.73 | 0.26 | 1096.59 | -6.67 | < .001 |
| Rating (joy) | -4.51 | 0.26 | 1096.59 | -17.37 | < .001 |
| Rating (masculine) | -1.69 | 0.26 | 1096.59 | -6.51 | < .001 |
| Rating (neutral) | -2.41 | 0.26 | 1096.59 | -9.27 | < .001 |
| Rating (sad) | -2.16 | 0.26 | 1096.59 | -8.33 | < .001 |
| Rating (surprised) | -3.90 | 0.26 | 1096.59 | -15.02 | < .001 |
| Age (young) | -3.68 | 0.26 | 1097.44 | -14.23 | < .001 |
| Rating (disgust) * age (young) | 1.42 | 0.37 | 1096.59 | 3.89 | < .001 |
| Rating (fear) * age (young) | 3.39 | 0.37 | 1096.59 | 9.27 | < .001 |
| Rating (feminine) * age (young) | 3.76 | 0.37 | 1096.59 | 10.28 | < .001 |
| Rating (joy) * age (young) | 6.45 | 0.37 | 1096.59 | 17.63 | < .001 |
| Rating (masculine) * age (young) | 3.42 | 0.37 | 1096.59 | 9.33 | < .001 |
| Rating (neutral) * age (young) | 4.41 | 0.37 | 1096.59 | 12.06 | < .001 |
| Rating (sad) * age (young) | 2.57 | 0.37 | 1096.59 | 7.03 | < .001 |
| Rating (surprised) * age (young) | 3.99 | 0.37 | 1096.59 | 10.90 | < .001 |

**Table S3b. Full Linear Mixed-Effects Model for Study**

**Supplemental Materials 4**

| **Random Effects** | | |
| --- | --- | --- |
| **Groups Name** | **Variance** | **Std. Dev** |
| Participant (intercept) | 0.22 | 0.46 |
| Image | 0.01 | 0.10 |
| Residual | 2.52 | 1.59 |
| Number of obs: 32652, groups: image, 56; participants, 66 | | |

| **Fixed Effects** | | | |  |  |
| --- | --- | --- | --- | --- | --- |
|  | **Estimate** | **Std. Error** | **t value** | **df** | **p-value** |
| Intercept | 3.40 | 0.07 | 47.82 | 142.80 | < .001 |
| Rating (disgust) | -0.19 | 0.05 | -3.64 | 31340 | .004 |
| Rating (fear) | -0.67 | 0.05 | -12.62 | 31340 | < .001 |
| Rating (joy) | -1.51 | 0.05 | -28.60 | 31340 | < .001 |
| Rating (sad) | 0.18 | 0.05 | 3.38 | 31340 | < .001 |
| Rating (neutral) | 0.64 | 0.05 | 12.17 | 31340 | < .001 |
| Rating (surprise) | -1.39 | 0.05 | -26.40 | 31340 | < .001 |
| Rating (feminine) | 0.32 | 0.05 | 6.06 | 31340 | < .001 |
| Rating (masculine) | 0.57 | 0.05 | 10.81 | 31340 | < .001 |
| Age (young) | -1.03 | 0.06 | -17.49 | 606 | < .001 |
| Rating (disgust) * age (young) | 0.36 | 0.07 | 4.76 | 31340 | < .001 |
| Rating (fear) * age (young) | 0.84 | 0.07 | 11.23 | 31340 | < .001 |
| Rating (joy) * age (young) | 1.03 | 0.07 | 13.78 | 31340 | < .001 |
| Rating (sad) * age (young) | 1.93 | 0.07 | 25.93 | 31340 | < .001 |
| Rating (neutral) * age (young) | 1.04 | 0.07 | 13.87 | 31340 | < .001 |
| Rating (surprise) * age (young) | 1.16 | 0.07 | 15.51 | 31340 | < .001 |
| Rating (masculine) * age (young) | 0.47 | 0.07 | 6.30 | 31340 | < .001 |
| Rating (feminine) * age (young) | 1.13 | 0.07 | 15.19 | 31340 | < .001 |

**Table S4. Full Linear Mixed-Effects Model for Study 2**

**Supplemental Materials 5**

| **Random Effects** | | | |
| --- | --- | --- | --- |
| **Groups Name** | **Variance** | **Std. Dev** | **Cor** |
| Participant (intercept) | 0.01 | 0.11 |  |
| Image | 0.00 | 0.02 |  |
| Age | 0.00 | 0.04 | -0.17 |
| Residual | 0.02 | 0.14 |  |
| Number of obs: 4200, groups: image, 575; participants, 42 | | | |

| **Fixed Effects** | | | |  |  |
| --- | --- | --- | --- | --- | --- |
|  | **Estimate** | **Std. Error** | **t value** | **df** | **p-value** |
| Intercept | 0.21 | 0.02 | 10.80 | 56.43 | < .001 |
| Rating (fear) | -0.01 | 0.01 | -0.46 | 521.91 | .647 |
| Rating (joy) | 0.19 | 0.01 | 17.16 | 518.53 | < .001 |
| Rating (neutral) | 0.05 | 0.01 | 4.45 | 521.75 | < .001 |
| Rating (sad) | 0.03 | 0.01 | 3.12 | 529.29 | .002 |
| Age (young) | 0.04 | 0.01 | 3.09 | 230.36 | .002 |
| Rating (fear) * age (young) | 0.02 | 0.02 | 1.17 | 517.19 | .242 |
| Rating (joy) * age (young) | 0.01 | 0.02 | 0.64 | 533.25 | .521 |
| Rating (neutral) * age (young) | 0.04 | 0.02 | 2.82 | 528.18 | .005 |
| Rating (sad) * age (young) | -0.01 | 0.02 | -0.56 | 519.98 | .576 |

***Table S4a. Linear mixed effects model for Study 3.***

| **Rating** | **Old Adult EMM** | **Young Old EMM** | **Estimate** | ***t* value** | ***p* value** |
| --- | --- | --- | --- | --- | --- |
| **Anger** | 0.21 | 0.25 | -0.04 | -3.09 | .002 |
| **Fear** | 0.20 | 0.26 | -0.06 | -4.58 | < .001 |
| **Joy** | 0.40 | 0.45 | -0.5 | -3.88 | < .001 |
| **Sad** | 0.24 | 0.27 | -0.03 | -2.43 | .02 |
| **Neutral** | 0.26 | 0.34 | -0.08 | -6.55 | < .001 |

***Table S5b. Pairwise comparisons for Study 3.***

| -0.14  -0.30  c’ = 0.48  c = 0.52  **Approach/Avoid Behavior**  **Negativity Index**  **Stimulus Age**  (Old = 0, young = 1) |
| --- |

***Figure S1. Mediation model evaluating whether stimulus-level emotional negativity mediates the relationship between stimulus age and approach/avoid behavior. Coefficients represent standardized betas.***
